# The Impact of a Nanocellulose-Based Wound Dressing in the Management of Thermal Injuries in Children: Results of a Retrospective Evaluation

**DOI:** 10.3390/life10090212

**Published:** 2020-09-19

**Authors:** Julia Cattelaens, Laura Turco, Luc M. Berclaz, Birgit Huelsse, Wolfgang Hitzl, Tobias Vollkommer, Karl J. Bodenschatz

**Affiliations:** 1 Clinic for Pediatric Surgery, Klinikum Nürnberg Süd, Breslauer Str. 201, 90471 Nuremberg, Germany; birgit.huelsse@klinikum-nuernberg.de (B.H.); karl.bodenschatz@klinikum-nuernberg.de (K.J.B.); 2 Max-Plank-Institute for Dynamics and Self-Organization, Am Fassberg 17, 37077 Göttingen, Germany; laura.turco@ds.mpg.de; 3 Paracelsus Medizinische Privatuniversität (PMU), Str. 1, 90419 Nuremberg, Germany; luc.berclaz@stud.pmu.ac.at; 4 Research Office, Biostatistics, Paracelsus Medizinische Privatuniversität (PMU), Strubergasse 21, 5020 Salzburg, Austria; wolfgang.hitzl@pmu.ac.at; 5 Department of Ophthalmology and Optometry, Paracelsus Medizinische Privatuniversität (PMU), Müllner Hauptstr. 48, 5020 Salzburg, Austria; 6 Research Program, Experimental Ophthalmology and Glaucoma Research, Paracelsus Medizinische Privatuniversität, Muellner Hauptstrasse 48, 5020 Salzburg, Austria; 7 Department of Oral and Maxillofacial Surgery, University Medical Center Hamburg-Eppendorf, Martinistraße 52, 20246 Hamburg, Germany; t.vollkommer@uke.de

**Keywords:** bacterial nanocellulose, wound dressing, burns, pediatric burns

## Abstract

The aim of this retrospective study is to evaluate for the first time the impact of a nanocellulose-based wound dressing in the treatment of pediatric patients with both partial- and deep-thickness burns. Usability and effectiveness were defined based on parameters such as frequency of dressing changes under narcosis, duration of hospital stay, onset of complications, need for additional treatments, and follow up scar formation assessment. Fifty-six children who sustained burns in the year 2018 and were treated with a nanocellulose-based wound dressing were included in the trial. The mean stay in hospital was 6.7 days. Patients underwent dressing changes under narcosis 2.4 times on average, and none had wound-associated infection. In all, 82% of the patients were treated only with nanocellulose-based wound dressings, and reepithelialization occurred after ten days. The majority of patients had scars with normal pigmentation (98%), vascularization (91%), height (92%), and pliability (92%). In conclusion, using a nanocellulose-based wound dressing for the treatment of both superficial, partial-thickness and deep, full-thickness burns has several advantages. Compared with the results published in literature on other wound dressings, it requires a moderate number of dressing changes under narcosis and results in short hospital stays. Additionally, it has a low associated infection rate and promotes wound healing.

## 1. Introduction

Thermal injuries in children lead to severe trauma with the possible need of long-term therapy and an unfavorable progression of scar formation, which has a significant influence on their physical and psychosocial development. In 2018, more than 2500 children with a median age of 3 years and an average total burned body surface of 6% [1] were treated in German burn centers [2].

The goals in the management of thermal injuries in pediatric patients according to the AWMF (Arbeitsgemeinschaft der wissenschaftlich medizinischen Fachgesellschaften e.V.) are avoiding frequent dressing changes, reducing pain, preventing wound infections, improving patient comfort, and promoting healing [2].

Generally, burn wound management depends on the severity of suffered burns. Superficial, partial-thickness burn wounds up to grade 2a are treated applying skin substitutes or dermal equivalents [2,3,4,5] and usually take two to three weeks to heal completely [6]. Deep, full-thickness dermal skin burns of degree 2b–3 require excision, or enzymatic debridement, a temporary coverage of the open wound, and grafting. Deep, full-thickness burn regeneration takes more than three weeks, and it is often associated with scarring unless grafted [7]. Since burn depths are often mixed and early discrimination between the two types by clinical observation is not always straightforward, an ideal skin substitute, which could facilitate healing of both superficial, partial-thickness and deep, full-thickness burns, before grafting, seems to be required.

In the last two decades, several advancements have been made in developing synthetic wound dressings for the treatment of burns or skin graft donor sites. One of the latest products developed in this field is Epicite hydro^®^, a wound-coverage made of bacterial nanocellulose. Bacterial nanocellulose represents a promising biomaterial for wound dressings [8,9]. It is characterized by a nanofiber network structure that confers mechanical stability and flexibility [10,11]. It is highly biocompatible [12] and has a water content of at least 95% [13]. This supports long-term dermal hydration [12,14] and creates a moist environment, preventing excessive fluid loss through the wound [15] while exudate can be adsorbed. Epicite hydro^®^ has a soft texture, is non-adhesive, and semitransparent. Bacterial-nanocellulose-based dressings significantly reduce intradermal temperature and have a cooling effect, which is based on evaporation. The latest published literature shows in an ex vivo burn model that more viable cells were found in the immunofluorescence in burn injuries that received cooling treatments [16]. It has been shown that bacterial nanocellulose dressings can be easily soaked with antiseptic solutions such as polyhexanide, octenidine, or povidone iodine in order to release them from the wound [15,17,18], showing a potential application for clinical use. However, studies on the impact of Epicite hydro^®^, especially in pediatric burn wound management, are scarce.

In this study, a retrospective review was performed to investigate the impact of Epicite hydro^®^ on wound healing of thermal injures in pediatric patients, by evaluating the frequency of dressing changes, hospital stay duration, complications, the need for additional treatments, and scar formation.

## 2. Results

### 2.1. Patients

This study enrolled 56 children with a median age of 2.4 years who were treated with Epicite hydro^®^ from January to December 2018. Patient demographics are shown in Table 1. All patients were in normal healthy conditions and without comorbidities at the time of the accident. The majority of injuries occurred in a domestic environment (95%) and most of the patient sustained contact (48%) or scald burns (46%). The most affected areas were hands (63%) or arms (30%). Cold water was applied in 38 cases (69%) of all burn wounds. A mean of 3% of total body surface area burned (TBSA) with a range of 1–20% was estimated according to the Lund and Browder chart. Forty-three burn sites were considered as superficial, partial-thickness burns, while thirteen were determined to be deep, partial- or full-thickness burns.

### 2.2. Duration of Hospital Stay and Dressing Changes

The mean duration of hospital stay was 6.7 days (std. 1.1, range 3–20), the frequency of surgeries and dressing changes under narcosis was 2.5 times (std. 3.5, range 1–6) during the period of treatment. Patients with superficial, partial-thickness burns(SPTB) were hospitalized for 5.7 days, patients with deep, partial- or full-thickness burns (DTB) for 9.8 days (*p* = 0.002). The frequency of dressing changes under narcosis or analgosedation was 2.3 and 3.4 times, respectively. There was a significant difference between the two groups (*p* < 0.001) (Table 2).

### 2.3. Infection and Complications

None of the 56 patients had wound-associated infections, with signs of infections regarding the wounds or sepsis, while they were treated with Epicite hydro^®^. Wound swabs were collected after clinical assessment and tested. None of the 39 tested patients (70%) were diagnosed with multiresistant pathogens in their wounds. Antibiotics were not given on a regular basis. One patient developed pneumonia during his stay in hospital and was treated with antibiotics. Three patients sustained pain crises during the treatment with Epicite hydro^®^. The dressing was then completely removed and replaced with Suprathel^®^ (Table 2) (Polymedics Innovations GmbH, Heerweg 15D, 73770 Denkendorf, Deutschland).

### 2.4. Reepithelialization and Need for Further Treatments

Forty-six (82%) patients were treated only with Epicite hydro^®^. Reepithelialization occurred after the 10th day of treatment, so no additional treatment was required. Ten patients had to undergo further procedures. Three patients (5%) needed a further split-thickness skin graft, and four patients (7%) obtained full-thickness skin grafts. Cortisone was applied to three patients (5%). There was a significant difference between SPTB and DTB (*p* = 0.001) (Table 2).

### 2.5. Scar Formation, Pain, and Itchiness

The quality of scars was assessed in 54 patients, analyzing the whole group using the Vancouver Scar Scale (VSS), the median overall score was 0 (range 0–8). Two patients did not join the follow-up care in our hospital.

Six months post burn, most patients presented normal pigmentation (98%), normal vascularity (90%), normal-height scar (92%), and normal pliability (92%). There was a significant difference in regard to vascularization between the SPTB and DTB groups (*p* = 0.03) (Table 3).

## 3. Discussion

Epicite hydro^®^ is a newly developed, non-animal-derived wound dressing. In this study, we report our experience with Epicite hydro^®^ in the treatment of both SPTB and DTB in children. To the best of our knowledge, this is the only retrospective study investigating the long-term use of Epicite hydro^®^, providing information within the treatment period about frequency of dressing changes, duration of hospital stay, onset of complications, and need for additional treatments.

Epicite hydro^®^ was particularly easy to handle for clinical investigators in pediatric burn management and supported a long-term dermal hydration of the wound. It exhibited excellent adherence to the wound surface in different parts of the body. We hypothesize this outcome to be due to the properties of the bacterial-nanocellulose base of Epicite hydro^®^. The high mechanical stability and flexibility [9,10] allow the dressing to conform to the wound bed and adapt to patient movements without risk of detachment or rupture. In general, the adherence to the wound is related to the water holding/release capacity of the wound dressing, which has to be able to absorb large amounts of exudate. Otherwise, the wet layer between the wound and the dressing can cause detachment of the latter and a lag in healing process. This may occur especially if a wound dressing is applied to the burn in the first few days’ post injury, as observed in a clinical study employing Suprathel^®^ in donor sites of split-thickness skin grafts [3]. Moreover, a good adherence to the burn surface in children has been reported for Suprathel^®^ by Rashaan et al. [19]. However, they suggest that an extensive wound bed debridement is required in order to achieve good adherence of the dressing. In this study, there was no need for such an excisional debridement.

Epicite hydro^®^ has a high water content (95%) [14], which resulted in adhesion to the wound, even if applied early after the first surgical or enzymatic debridement. The hydrophilic properties [15] and the optimal vapor transmission rate (2900 g m^−2^ day^−1^) [12] of bacterial nanocellulose enable the uptake of water solutions, such as exudate, avoiding fluid accumulation. The physical properties of bacterial nanocellulose are also responsible for maintaining humidity and preventing wound dressing detachment, which would cause pain when exchanged. Indeed, renewing the Epicite hydro^®^ after application was not required over the duration of the treatment since no detachment from the wound bed occurred.

Regarding the frequency of dressing changes and duration of hospital stay, our outcome showed that patients with SPTB were hospitalized for 5.7 days and dressing was changed 2.3 times, while patients with DTB stayed in hospital for 9.8 days and changes occurred 3.4 times. In one study, employing a commonly used (semi) synthetic dressing (Suprathel^®^) for treatment of burns in children, a longer length of hospital stay (10 days) and slightly more frequent dressing changes were found [19]. Aboelnaga et al. reported a decrease in dressing changes and hospital stay when applying microbial cellulose to partial-thickness burns in comparison with applying silver sulfadiazine in a randomized trial [20]. These findings and our observations may show a potential advantage in using products based on bacterial cellulose, such as Epicite hydro^®^, in the management of SPTB, or even DTB, in children. However, a comparative study is needed to confirm this potentiality.

A desirable decrease in the frequency of dressing changes and duration of hospital stay contribute to an increase in patient comfort. In particular, the former reduces the pain, anxiety, and distress during application and removal of wound dressing. Additionally, we experience easy take off of Epicite hydro^®^, as described in other studies as well as reduced local pain [15,17]. This is, together with the associated early discharge, extremely beneficial for pediatric patients and their parents. Epicite hydro^®^ was as well particularly easy to handle for clinical investigators.

We believe that adherence of the dressing to the wound bed is a factor that influences the frequency of changes and duration of hospital stay. If good adhesion is achieved, adequate fluid balance is provided, as explained above. Consequently, the dressing protects the wound from excessive fluid loss, which has been reported to accelerate wound healing [15].

### 3.1. Infection and Complications

We observed no wound-associated infection or cases of sepsis. Similarly, others reported a low infection rate (8% of the population) [21] or none [3] when Suprathel^®^ is used. Schwarze et al. attribute this to the 80% water permeability of the porous membrane, which allows sufficient fluid drainage [3].

Epicite hydro^®^ has an even higher water content than Suprathel^®^ and a desirable water transmission rate that may decreases the bacterial contamination risk [12]. This may suggest that Epicite hydro^®^ is even more suitable in the prevention of infections. Although Picheth et al. mention that bacterial cellulose has no activity against bacterial infection [9], we and others observed favorable effects in this matter [12,22]. Additionally, de Mattos et al. proposed a simple and quick procedure to load Epicite hydro^®^ with polyhexanide, octenidine, or povidone-iodine-containing antiseptics [15,17] underlining the high suitability of such a wound dressing for clinical use.

Complications associated with the treatment of Epicite hydro^®^ happened in five percent of our 56 patients because of pain crises. In these three cases, the material had to be taken off as no other reason for the pain crisis could be found. The three patients were then treated with Suprathel^®^ instead. After consulting the producer, we decided to stop loading Epicite hydro^®^ with polyhexanide solution and instead use normal steriline. No pain crises were observed when the addition of polyhexanide was terminated. We do not have any explanation for that. The wound infection rates stayed as low as before, which seems to underline the antiseptic effect of the bacterial nanocellulose itself. In all other cases, pain relief was not necessary on a regular basis.

In our study, only one patient was diagnosed with pneumonia during hospital stay, which we attributed to the critical burn site, the ventral thorax, and to a possibly protracted infection of the upper respiratory tract.

### 3.2. Cost Effectiveness

The comparison of the cost effectiveness of Epicite hydro^®^ with Suprathel^®^ and two other commonly used wound dressings, Mepilex Ag^®^ and Acticoat^®^, based on the material costs, revealed that Epicite hydro^®^ may be the most cost efficient. A sheet of 10 × 10 cm of Epicite hydro^®^ costs about 17 Euro, while Suprathel^®^ and Acticoat^®^ cost 126 and 47 Euro, respectively. Moreover, the low number of dressing changes, length of hospitalization, treatment of infections, and the related nursing care costs, as shown in this work, may results in a reduction of total costs. This is an advantage even in comparison with the usage of Mepilex Ag^®^ (10 × 10 cm sheet cost is about 10 Euro). Even though Mepilex Ag^®^ is cheaper, it has been reported that the dressing is usually changed every 3–4 days [23], consequently increasing the costs within the whole treatment period.

### 3.3. Reepithelialization and Need for Further Treatments

The results regarding the reepithelialization rate showed that almost all patients with SPTB (98%) and 31% of children with DTB healed within the period of treatment (10 days). When wound healing was not reached within 10 days, children received additional treatments. In literature, studies on the wound management of partial-thickness burns in children with other (semi) synthetic dressings reported a similar time of reepithelialization (9.5 days to 12 days) [3,19,24]. Everett et al. had an average time of reepithelialization of 9.5 days and Rashaan et al. of 13 days with Suprathel^®^ [8,19].

Aboelnaga et al., 2018, showed a not significant but quicker epithelialization with cellulose-based materials compared to that with silver sulfadiazine [20]. Epicite hydro^®^ seems to have similar effects on reepithelialization compared to other skin substitutes for SPTB management. Rapid wound reepithelialization is crucial not only to prevent wound site infection but also to reduce the risk of hypertrophic scarring, which is rarely evaluated in the pediatric population [19]. Studies have shown that the risk of developing hypertrophic scars is low in burn injuries that heal within 21 days [6,25]. Consistently, as wounds treated with Epicite hydro^®^ healed within the optimal time period, we found a good scar quality according to the scar assessment 3–6 months post injury using the VSS. The median VSS height score was ≤1, indicating non-hypertrophic scars [26], with no significant difference between SPTB and DTB. This may imply that Epicite hydro^®^ can be used for both superficial and deep burn management with excellent results in term of wound healing and favorable scar formation.

### 3.4. Limitations

A limitation of this study is its retrospective nature and the small sample size. This may have contributed to the low detection of significant differences. Another limitation is the absence of pain assessment via the Visual Rating Scale, which would allow the collection of systematic data to distinguish between pain, anxiety, and distress.

A larger, prospective, randomized trial would be significant to confirm the results of this study on the usability and the effectiveness of Epicite hydro^®^ for burn management in pediatric patients. A comparative study regarding differences between other synthetic wound dressings, especially the frequently used Suprathel^®^, should be performed.

## 4. Materials and Methods

This study was approved by the department of Pediatric Surgery at the Nuremberg General Hospital. Review board number: IRB-2019-015.

### 4.1. Patients

From January to December 2018, we treated n = 56 pediatric patients up to the age of 15 years with sustained flame, scald, explosion, or contact burns. All burns apart from facial ones were treated with Epicite hydro^®^. There was no limitation in the estimated total body surface area burned. There was no randomization in deciding whether or not to treat the patient with Epicite hydro^®^. There were no exclusion criteria, and all of the patients treated with Epicite hydro^®^ were enrolled in this study.

### 4.2. Data

The following data were collected in individual files for each patient: age, gender, percentage of total body surface area burned (TBSA %), time and place where the injury occurred, time of admission, degree, site, and cause of burn. The burn depth was assessed by clinical evaluation before debridement in the first 24–48 h after injury and every time the dressing was changed. Wound depths were scaled after clinical findings and graded after international guidelines [2]. Wounds of grade 2a were then categorized as superficial, partial-thickness burns (SPTB) and wounds of grade 2b–3 as deep, partial- or full-thickness burns (DTB) with high risk of scar formation and possible skin graft treatment. TBSA% was estimated using the Lund and Browder chart [27].

Clinical parameters of our study included frequency of wound debridement and dressing changes performed under general narcosis, length of hospitalization, complications such as infectious rates or pain crises, and number of needed skin grafts. Long-term scar formation was accessed by specialists three to six months post-burn using the Vancouver Scar Scale (VSS) [26,28] and documented with photographs. VSS is based on the observation of four parameters: pigmentation, vascularity, pliability, and scar height. Pigmentation was scored on a 0–2 points scale, vascularity and scar height on a 0–3 points scale, and pliability on a 0–5 points scale, as shown in Table 4. The sum of the scores resulted in a number that indicates whether the scar is hypertrophic. As Thompson et al. determined in a survey, a score ≥1 is highly sensitive and specific for the diagnosis of a hypertrophic scar formation [28]. We used this value as the cutoff.

### 4.3. Treatment Protocol

**Figure 1:**
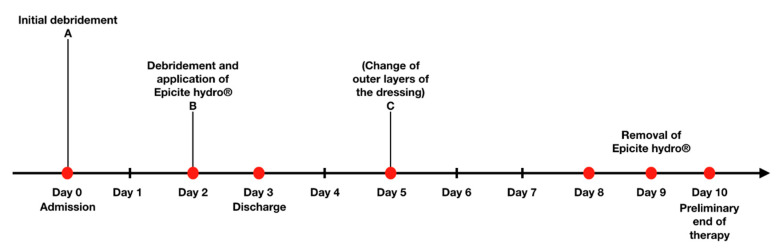
Timeline of the treatment: Day 0, initial debridement; Day 2, debridement and application of Epicite hydro^®^; Day 9, removal of Epicite hydro^®^**.**

The treatment protocol (Figure 1) is attributable to the underlying experience acquired during the time using Epicite hydro^®^ in our clinic. Its goal is to optimize the treatment with Epicite hydro^®^ in burned children according to the guidelines of the AWMF [2]. Patients were admitted following the same guidelines.

**Figure 2:**
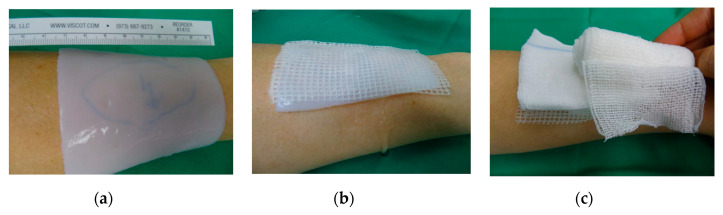
Epicite hydro^®^ is applied on the debrided burn wound (**a**) and covered with a layer of Vaseline gauze (**b**) and a moistened and a dry compress (**c**). The dressing is then fixed (**c**).

According to the treatment protocol, wounds were cleaned by superficial debridement upon admission (Day 0). Superficial debridement consists of a standard removal of loose skin remnants and open blisters in analgosedation. The wound dressings were then fixed with polyhexanide-gel and covered with a Mepilex^®^ grid and moistened compresses. In the first 48 h after admission (Day 2), patients underwent a surgical debridement by using moistened compresses or surgical spoon to remove the eschar or an enzymatic debridement with Nexobrid^®^, if the injury was suspected to be DTB. Surgical debridement or enzymatic debridement were both performed under general narcosis. Microbiological reliable tests were performed only for 39 patients. After evaluating the retrospective data and noticing that swabs were not taken of all patients on a regular basis, we clarified our protocol, so that every patient got a swab, before putting on Epicite hydro^®^, and to get higher data quality. Wound photographs were taken. Before application, Epicite hydro^®^ was soaked in polyhexanide solution and later on in normal steriline and then cut into suitable dimensions to fully cover the burned area (1 cm exceeding the wound size). Epicite hydro^®^ was then covered with a double layer of Vaseline gauze followed by one moistened and one dry dressing (Figure 2). Depending on the extent of the burn, patients were admitted to the ward or discharged and seen in the outpatient clinic. Epicite hydro^®^ was then left in situ for seven days.

In the initial stage of the trial with no prior usage in the clinic, the outer layers of the dressing were changed three days after Epicite hydro^®^ was applied to the wound (around Day 5) Epicite hydro^®^ was left in place, covered with a double Vaseline gauze and dry compresses. Since no complications occurred and more experience with the new material was acquired, the outer layer dressing change around day five was not done anymore to reduce dressing changes.

Between Days 8 and 10, Epicite hydro^®^ was soaked in normal steriline and then removed. The duration of the treatment was set to a maximum of 10 days to verify the impact of Epicite hydro^®^ on wounds within the general healing time for SPTB (10–14 days) [6] and within a period in which grafting of DTB was still possible. At the end of the treatment, wound assessment was performed by experienced burn specialists. It was decided whether a skin graft or, in case adequate wound healing had occurred, a dexpanthenol ointment therapy combined with a long-term compression therapy were needed.

### 4.4. Statistical Methods

Data consistency was checked, and data were screened for outliers. Descriptive data are presented as means or medians. Fisher’s exact and Pearson’s chi-squared test was used to analyze the cross-tabulation table; Student t-tests were used to analyze continuously distributed variables. All tests were two-sided with a significance level of 5%. All statistical analyses were performed with the help of STATISTICA 13 (Hill, T. and Lewicki, P. Statistics: Methods and Applications. Stat Soft, Tulsa, OK, USA).

## 5. Conclusions

The results of this study suggest an excellent impact of Epicite hydro^®^ on the treatment of thermal injuries in children. According to our experience, it was very easy to handle; exhibited excellent hydration and adherence to the wound surface, without need for extensive wound debridement; and required few dressing changes. No wound-associated infections, minimal complications, and rapid reepithelialization were observed, allowing an early discharge after application. Moreover, good wound healing and no hypertrophic scars were observed in both treated SPTB and DTB. Our study illustrates that this new skin substitute represents a potential good wound dressing for the treatment of burns or skin graft donor sites.

## Figures and Tables

**Table 1:** Patient demographics, total body surface area (TBSA, %), SPTB = superficial, partial-thickness burn, DTB = deep, partial- or full-thickness burn.

Age (Years), Mean (Std., Range)	2.4 (2.89, 0–15)
Gender, n (%)	
Male	40 (71)
Female	16 (29)
Location of the accident, n (%)	
Domestic	53 (94.6)
School/Kindergarten	2 (3.6)
Child abuse	1 (1.8)
Etiology of burn, n (%)	
Scald	26 (46.4)
Contact	27 (48.2)
Explosion	2 (3.6)
Flame	1 (1.8)
Site of burn, n (%)	
Arms	17 (30.5)
Hands	35 (62.5)
Legs	7 (12)
Feet	9 (16)
Head	7 (12.5)
Neck	3 (5.4)
Thorax (ventral)	9 (16.1)
Thorax (dorsal)	2 (3.6)
%TBSA, mean (std., range)	
2a degree of burn (n = 51)	2.9 (3.15, 1–20)
2b degree of burn (n = 7)	1.9 (1.86, 1–6)
3 degree of burn (n = 8)	1 (0.0, 1–1)
Depth of burn, n (%)	
SPTB	43 (77)
DTB	13 (23)

**Table 2:** Overview of study parameters (numbers of surgeries or dressing changes under narcosis, complications, additional treatments). SPTB = superficial, partial-thickness burn, DTB = deep, partial- or full-thickness burn.

Parameters	SPTB (n = 43)	DTB (n = 13)	*p*-Value
Number of surgeries	2.3 (0.93)	3.4 (1.39)	0.002
mean (std.)			
Duration of hospital stay (days)	5.7 (2.24)	9.8 (5.0)	0.001
mean (std.)			
Complications, n (%)			
Pain crisis	1 (2.3)	2 (15.4)	0.13
Pneumonia	1 (2.3)	0 (0)	1.0
Burn-associated infection	0 (0)	0 (0)	1.0
Additional treatments, n (%)	1 (2.3)	9 (69)	0.001

**Table 3:** Vancouver Scar Scale parameters and score 3–6 months post burn. SPTB = superficial, partial-thickness burn, DTB = deep, partial- or full-thickness burn.

Parameters	SPTB n = 42	DTB n = 12	*p*-Value
Pigmentation, (points), n (%)	0:42 (0)	0:11 (91.6)	0.22
	1:0 (0)	1:1 (8.3)	
	2:0 (0)	2:0 (0)	
Vascularity, (points), n (%)	0:40 (95.2)	0:9 (75)	0.03
	1:1 (2.4)	1:3 (25)	
	2:1 (2.4)	2:0 (0)	
	3:0 (0)	3:0 (0)	
Scar height, (points), n (%)	0:40 (95.2)	0:10 (83.4)	0.11
	1:2 (4.8)	1:1 (8.3)	
	2:0 (0)	2:1 (8.3)	
	3:0 (0)	3:0 (0)	
Pliability, (points), n (%)	0:40 (95.2)	0:10 (83.4)	0.14
	1:0 (0)	1:1 (8.3)	
	2:2 (4.8)	2:1 (8.3)	
	3:0 (0)	3:0 (0)	
	4:0 (0)	4:0 (0)	
	5:0 (0)	5:0 (0)	
Vancouver Scar scale: cutoff ≥ 1 point, n (%)	0:38 (91)	0:8 (67)	0.14
	1:1 (2)	1:2 (17)	
	2:1 (2)	2:1 (8)	
	5:2 (5)	5:0 (0)	
	8:0 (0)	8:1 (8)	

**Table 4:** The Vancouver Scar Scale.

Vancouver Scar Scale	Pigmentation	Vascularity	Scar Height (mm)	Pliability
0	Normal	Normal	Flat	Normal
1	Hypopigmentation	Pink	<2	Soft
2	Hyperpigmentation	Red	2–5	Retreating
3	-	Purple	>5	Hard/stiff
4	-	-	-	Scar-strand
5	-	-	-	Contracture

## Abbreviations

AWMF	Arbeitsgemeinschaft der wissenschaftlich medizinischen Fachgesellschaften e.V
DTB	Deep, partial- or full-thickness burn
SPTB	Superficial, partial-thickness burn
TBSA	Total body surface area (%)
VSS	Vancouver Scar Scale
